# Biphasic Regulation of Mesenchymal Genes Controls Fate Switches During Hematopoietic Differentiation of Human Pluripotent Stem Cells

**DOI:** 10.1002/advs.202001019

**Published:** 2020-08-19

**Authors:** Hongtao Wang, Mengge Wang, Yuqi Wen, Changlu Xu, Xiaoyuan Chen, Dan Wu, Pei Su, Wen Zhou, Tao Cheng, Lihong Shi, Jiaxi Zhou

**Affiliations:** ^1^ State Key Laboratory of Experimental Hematology National Clinical Research Center for Blood Diseases Institute of Hematology & Blood Diseases Hospital Chinese Academy of Medical Sciences & Peking Union Medical College Tianjin 300020 China; ^2^ CAMS Center for Stem Cell Medicine PUMC Department of Stem Cell and Regenerative Medicine Tianjin 300020 China; ^3^ School of Basic Medical Science and Cancer Research Institute Central South University Changsha 410013 China

**Keywords:** HAND1, hematopoietic differentiation, human pluripotent stem cells, mesenchymal genes, SNAI1

## Abstract

Epithelial‐mesenchymal transition (EMT) or its reverse process mesenchymal‐epithelial transition (MET) occurs in multiple physiological and pathological processes. However, whether an entire EMT–MET process exists and the potential function during human hematopoiesis remain largely elusive. Utilizing human pluripotent stem cell (hPSC)‐based systems, it is discovered that while EMT occurs at the onset of human hematopoietic differentiation, MET is not detected subsequently during differentiation. Instead, a biphasic activation of mesenchymal genes during hematopoietic differentiation of hPSCs is observed. The expression of mesenchymal genes is upregulated during the fate switch from pluripotency to the mesoderm, sustained at the hemogenic endothelium (HE) stage, and attenuated during hemogenic endothelial cell (HEP) differentiation to hematopoietic progenitor cells (HPCs). A similar expression pattern of mesenchymal genes is also observed during human and murine hematopoietic development in vivo. Wnt signaling and its downstream gene SNAI1 mediate the up‐regulation of mesenchymal genes and initiation of mesoderm induction from pluripotency. Inhibition of transforming growth factor‐*β* (TGF‐*β*) signaling and downregulation of HAND1, a downstream gene of TGF‐*β*, are required for the downregulation of mesenchymal genes and the capacity of HEPs to generate HPCs. These results suggest that the biphasic regulation of mesenchymal genes is an essential mechanism during human hematopoiesis.

## Graphical abstract

Herein, biphasic regulation of mesenchymal genes is found during hematopoietic development at the transcriptomic level. The dynamic expression of mesenchymal genes mediated by SNAI1 and HAND1 is essential for the fate switches during human hematopoiesis. These findings provide insights into the molecular mechanisms governing early human hematopoietic development.

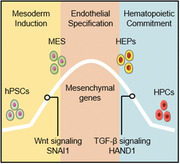

## 1 Introduction

Human pluripotent stem cells (hPSCs) provide a tractable system to analyze human hematopoietic development given their capacity to undergo long‐term self‐renewal and to differentiate into all three germ layers.^[^ ^1^ ^]^ Although hematopoietic stem cell (HSC) transplantation and transfusion of functional blood cells are effective for treating diverse blood disorders, the shortage of donors can limit these applications.^[^ ^2^ ^]^ Hematopoietic differentiation of hPSCs has emerged as a promising strategy to generate therapeutic cells. Despite recent advances in the induction of hPSC differentiation, the production of HSCs with engraftment potential from hPSCs remains highly challenging.^[^ ^3^ ^]^ Thus, elucidating the mechanisms underlying hPSC hematopoietic differentiation will provide insights into early human hematopoietic development and may also benefit the production of HSCs and functional blood cells from hPSCs for clinical applications.

Epithelial‐mesenchymal transition (EMT) or its reverse process mesenchymal‐epithelial transition (MET) occurs in multiple physiological and pathological processes, including mammalian development and malignant progression.^[^ ^4^, ^5^ ^]^ Recent studies demonstrated that transition to a mesenchymal state is a key mechanism for cell fate decisions during programming or reprogramming. For example, MET is essential for somatic cell reprogramming to a pluripotent state,^[^ ^6^, ^7^ ^]^ while the initiation of earlier EMT prior to MET facilitates iPSC generation from fibroblasts.^[^ ^8^ ^]^ During hematopoietic development or leukemic transformation, the role of EMT and its modulators have been investigated.^[^ ^9^, ^10^ ^]^ Twist1 regulates embryonic hematopoietic differentiation by directly targeting Myb and Gata2.^[^ ^11^ ^]^ Twist1 is highly expressed in leukemia stem cells and promotes tumor cell growth.^[^ ^12^, ^13^ ^]^ The deletion of Zeb2 severely impairs the differentiation of murine embryonic cells and adult hematopoietic stem/progenitor cells.^[^ ^14^, ^15^ ^]^ Although these EMT regulators play essential roles in distinct temporal windows of hematopoiesis or hematological malignancies, whether an entire EMT–MET process occurs and functions during hematopoiesis remain largely elusive.

Hematopoietic differentiation of hPSCs recapitulates hematopoiesis in vivo and proceeds through three main stages: mesoderm specification, hemogenic endothelial cell (HEPs) emergence and endothelial to hematopoietic transition (EHT) to produce hematopoietic cells.^[^ ^16^ ^]^ Each stage is regulated by extrinsic factors, intracellular signaling pathways, and transcription factors. Previous studies demonstrated that BMP4 promotes EMT and mesoderm differentiation by regulating the EMT transcription factors MSX2 and SLUG.^[^ ^17^, ^18^ ^]^ The EMT transcription factor SNAI1 controls gastrulation and mesoderm commitment.^[^ ^19^, ^20^, ^21^ ^]^ In addition, during EHT, inhibition of transforming growth factor‐*β* (TGF‐*β*) signaling facilitates hematopoietic cell generation,^[^ ^22^, ^23^, ^24^ ^]^ which initiates EMT.^[^ ^25^ ^]^ These findings led us to propose that a sequential EMT–MET process might occur during hematopoiesis and underlie hematopoietic fate decisions. Thus, it is of great interest to identify key EMT regulators controlling hematopoietic differentiation and their crosstalk with the signaling pathways known to regulate cell fate transitions.

In this study, we discovered biphasic activation of distinct mesenchymal genes during hematopoietic differentiation of hPSCs and also found a similar expression pattern of mesenchymal genes during human and murine hematopoietic development in vivo. Mechanistically, we identified specific mesenchymal genes that collaborate with Wnt and TGF‐*β* signaling to control fate switches during different temporal windows of hPSC hematopoietic differentiation. Thus, our findings provide novel insight into the mechanisms underlying human hematopoietic development and should benefit the production of HSCs and functional blood cells from hPSCs for clinical applications.

## 2 Results

### 2.1 Biphasic Regulation of Mesenchymal Genes During Hematopoietic Differentiation of hPSCs

**Figure 1:**
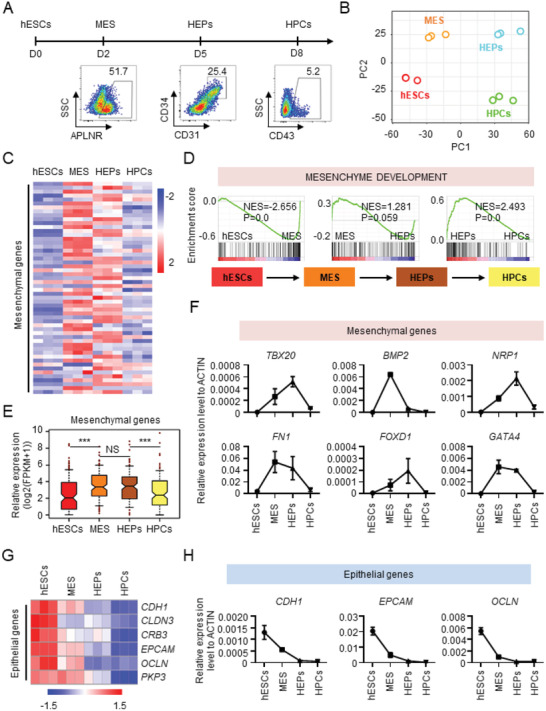
Biphasic regulation of mesenchymal genes during hematopoietic differentiation of hPSCs. A) Schematic overview of hESC hematopoietic differentiation in the chemically defined system. B) PCA results show a clear stepwise fate switches from pluripotent cells to HPCs. C) The heatmap showing different expression of mesenchymal genes between the four populations of cells during hematopoietic differentiation of hESCs. D) The enrichment of “Mesenchyme development” gene set between the four populations of cells during hematopoietic differentiation of hESCs. E) The dynamic expression of mesenchymal genes between the different populations of cells derived from hESCs during hematopoietic differentiation. F) The dynamic expression of characteristic mesenchymal genes in the four populations of cells during hematopoietic differentiation of hESCs, as detected with real‐time PCR. G) The heatmap showing down‐regulation of epithelial genes during hematopoietic differentiation of hESCs. H) The expression of characteristic epithelial genes in the four populations of cells during hematopoietic differentiation of hESCs, as detected with real‐time PCR. NS, not significant and ***P < 0.001.

Human hematopoiesis can be modeled using coculture systems with stromal cells or chemically‐defined culture conditions.^[^ ^26^, ^27^, ^28^ ^]^ Different populations of differentiated cells, including APLNR^+^ mesoderm cells, CD31^+^CD34^+^ HEPs, and CD43^+^ hematopoietic progenitor cells (HPCs), were generated sequentially after 7–8 days of hPSC differentiation (**Figure** **1**A). To identify molecular machinery underlying cell‐fate switches, we conducted a genome‐wide transcriptomic analysis of each cell type during this process. Principle component analysis (PCA) revealed stepwise fate switches from pluripotent cells to HPCs (Figure 1B). Genes associated with pluripotency (PL), mesoderm (MES), HEPs, and HPCs were enriched in each cell population at the respective stages (Figure S1A, Supporting Information). Furthermore, the expression of established marker genes, including *NANOG*, *MESP1*, *SOX7*, and *GATA1* for each stage was validated using quantitative real time polymerase chain reaction (qRT‐PCR) analysis (Figure S1B, Supporting Information). Thus, hematopoietic differentiation from hPSCs occurs in a stepwise fashion and recapitulates the developmental process in vivo.

To identify differentially expressed genes between each cell population, we conducted Gene Set Enrichment Analysis (GSEA), which revealed a cohort of mesenchymal genes with differential expression between the four populations of cells during differentiation (Figure 1C,D). 183 mesenchymal genes were identified based on a mesenchymal development gene set from the GSEA database, and their differential expression between distinct cell populations was determined to be significant (Figure 1E, hESCs vs MES, *P *< 0.001; HEPs vs HPCs, *P *< 0.001).

Analysis of the temporal expression of mesenchymal genes revealed a dynamic, biphasic pattern. The expression of mesenchymal genes was upregulated during the fate switch from pluripotency to mesoderm and sustained at the hemogenic endothelium (HE) stage, but was downregulated during HEP differentiation to HPCs (Figure 1E). We analyzed representative mesenchymal genes (*TBX20*, *BMP2*, *NRP1*, *FN1*, *FOXD1*, and *GATA4*) by qRT‐PCR, which confirmed the dynamic expression pattern (Figure 1F). To exclude the possibility that the dynamics of mesenchymal gene expression is cell‐line specific, we utilized BC1 cells, a widely used human iPSC line,^[^ ^26^, ^29^ ^]^ which revealed an indistinguishable temporal pattern (Figure S1C, Supporting Information). The same expression pattern of mesenchymal genes was detected with hESCs undergoing hematopoietic differentiation with the OP9 coculture system using previously reported RNA‐seq data^[^ ^30^ ^]^ (Figure S1D, Supporting Information). Thus, a cohort of mesenchymal genes are expressed in a biphasic pattern during hematopoietic differentiation of hPSCs.

To determine whether an entire EMT–MET process occurred during hematopoietic differentiation, we quantified the expression dynamics of epithelial genes. The epithelial genes were expressed at the highest level in hPSCs and were gradually downregulated during the fate switches from mesoderm to HEPs and HEPs to HPCs (Figure 1G). Minimal expression of epithelial genes was observed in HEPs and HPCs. We also quantified epithelial gene expression in BC1 and the cells differentiated under the OP9 coculture condition, which revealed a similar expression pattern (Figure S1E,F, Supporting Information). This expression pattern was confirmed by qRT‐PCR analysis of epithelial gene expression (*CDH1*, *EPCAM*, and *OCLN*) (Figure 1H). Thus, in contrast to the biphasic expression pattern of mesenchymal genes, the epithelial genes showed a gradually decreased pattern of expression during hematopoietic differentiation of hPSCs.

### 2.2 Biphasic Regulation of Mesenchymal Genes Occurs During Hematopoietic Development In Vivo

**Figure 2:**
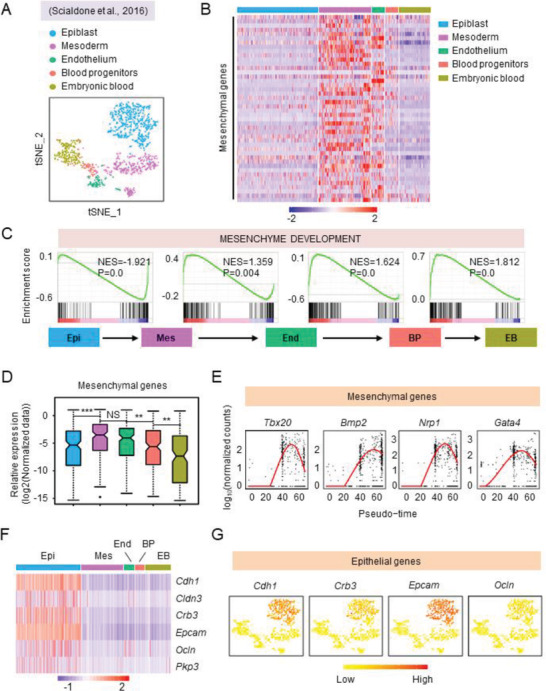
Biphasic regulation of mesenchymal genes occurs during hematopoietic development in vivo. A) t‐SNE showing five different developmental stages during embryonic hematopoiesis (E6.0‐E7.75) in vivo. B) The heatmap showing different expression of mesenchymal genes in five different populations during mouse embryonic hematopoiesis in vivo. C) The comparison of “mesenchyme development” gene expression between the cell populations from each stage of mouse embryonic hematopoiesis, as assessed by GSEA. D) The dynamic expression of mesenchymal genes at different stage of mouse embryonic hematopoiesis. E) The dynamic expression of representative mesenchymal genes during mouse embryonic hematopoiesis by pseudotime analysis. F) The heatmap showing the expression of epithelial genes during mouse embryonic hematopoiesis in vivo. G) t‐SNE of characteristic epithelial genes during mouse embryonic hematopoiesis in vivo. Epi, Epiblast, Mes, Mesoderm, End, Endothelium, BP, Blood progenitors, EB, embryonic blood. NS, not significant and **P < 0.01 and ***P < 0.001.

We asked whether the biphasic pattern also characterizes hematopoietic development in vivo. We utilized previously published single‐cell profiling data from early mouse embryos.^[^ ^31^ ^]^ Cells were reclassified into five populations, including epiblasts, mesoderm, endothelium, blood progenitors, and embryonic blood cells, based on the t‐distributed stochastic neighbor embedding (tSNE) analysis, which demonstrated a distinct distribution of the five cell populations with minimal overlap (**Figure** **2**A). Hallmark genes representing the distinct cell types were enriched in five populations, respectively (Figure S2A,B, Supporting Information). Mesenchymal genes were highly expressed in mesoderm and endothelium cells, but not in other cell populations (Figure 2B). Gene ontology (GO) term analysis for the stage‐specific genes revealed mesenchymal cell differentiation and mesenchymal development to be enriched terms (Figure S2C, Supporting Information). We compared the expression pattern of mesenchymal genes between different stages and found that the differential expression of mesenchymal genes between distinct cell stages was significant (Figure 2C,D).

We quantified the dynamics of mesenchymal gene expression during hematopoietic differentiation in mouse embryos and discovered the upregulation during the switch from pluripotency to the mesoderm, sustained high‐level expression from mesoderm to endothelium, and downregulation during the endothelium to blood progenitor transition. The pattern resembled that during hPSC hematopoietic differentiation (Figure 2C,D). The expression of representative mesenchymal genes (*Tbx20*, *Bmp2*, *Nrp1*, and *Gata4*) followed this dynamic pattern (Figure 2E; and Figure S2D, Supporting Information).

We also examined the expression pattern of mesenchymal genes during human hematopoietic development in vivo by taking advantage of the recently reported single‐cell profiling data obtained from human embryos.^[^ ^32^ ^]^ Specifically, we combined the datasets of endothelial cells (ECs) and hematopoietic stem and progenitor cells (HSPCs) and used them for further analysis (Figure S2E, Supporting Information). Feature genes representing the distinct cell types were enriched in the respective population (Figure S2F, Supporting Information). When compared with ECs, HSPCs exhibited a significant downregulation of mesenchymal gene (Figure S2G, Supporting Information). Thus, a biphasic regulation of mesenchymal gene expression occurs during hematopoietic development both in vitro and in vivo.

Finally, we quantified the expression of epithelial genes using data from the in vivo analyses. While epiblast cells exhibited intermediate expression levels of epithelial genes, minimal expression was detected in endothelium and embryonic blood progenitor cells (Figure 2F,G). These observations are consistent with the results from the in vitro experiments and suggest that an EMT to MET process does not occur in vivo.

### 2.3 Wnt Signaling is Essential for Mesenchymal Gene Expression and Induction of Differentiation

**Figure 3:**
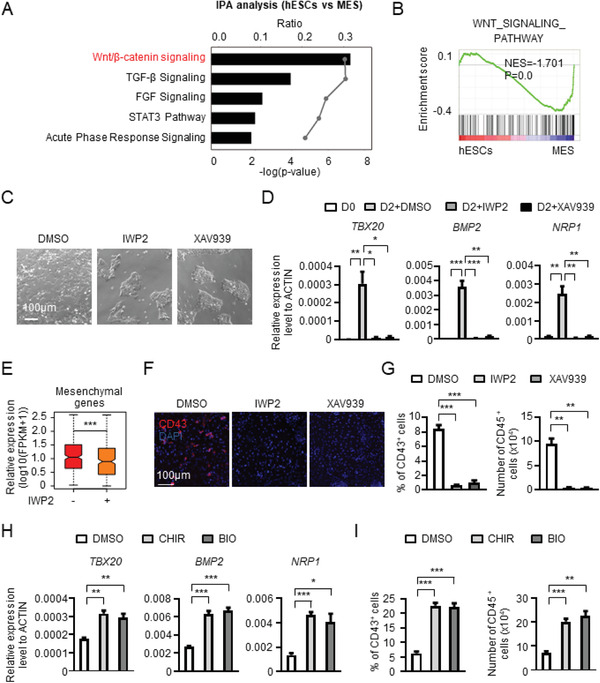
Wnt Signaling is essential for mesenchymal gene expression and induction of differentiation. A) IPA was performed to identify the underlying signaling from pluripotency to mesoderm and Wnt/*β*‐catenin signaling was the most activated signaling. B) GSEA of “Wnt signaling pathway” gene set between hESCs and mesoderm cells. C) The morphology of cells at day 2 of hematopoietic differentiation with or without the treatment of IWP2 and XAV939, inhibitors of Wnt signaling. D) The expression of representative mesenchymal genes with or without Wnt signaling inhibitors treatment detected by real‐time PCR. E) The box plot showing the expression of mesenchymal genes with or without IWP2 treatment. F) The immunofluorescence of CD43^+^ HPCs with or without Wnt signaling inhibitor treatment. G) The percentage of CD43^+^ HPCs (left) and the number of CD45^+^ HPCs (right) with or without Wnt signaling inhibitor treatment analyzed with flow cytometry. H) The expression of representative mesenchymal genes with or without the treatment of CHIR99021 (CHIR) and BIO analyzed with real‐time PCR. I) The percentage of CD43^+^ HPCs (left) and the number of CD45^+^ HPCs (right) with or without CHIR and BIO treatment analyzed with flow cytometry. *P < 0.05, **P < 0.01, and ***P < 0.001.

The dynamic expression pattern of mesenchymal genes during hematopoietic differentiation suggested that this constitutes a mechanism important for cell fate switches that control hPSC hematopoietic differentiation. We conducted ingenuity pathway analysis (IPA) of the RNA‐seq data and focused on the temporal window during the pluripotency to mesoderm transition. Wnt/*β*‐catenin signaling emerged as the top activated signaling pathway in this window (**Figure** **3**A). Consistent with this finding, GSEA revealed statistically significant enrichment of Wnt signaling‐linked genes in mesoderm cells versus pluripotent stem cells in vitro and in vivo (Figure 3B; and Figure S3A, Supporting Information).

Based on the temporal pattern of Wnt signaling activation during pluripotency to mesoderm transition, we tested whether it is essential for the upregulation of mesenchymal genes. We utilized two small‐molecule Wnt signaling inhibitors, IWP2 and XAV939^[^ ^33^, ^34^ ^]^ to evaluate the relationship between Wnt signaling, mesenchymal gene expression, and hPSC differentiation. The inhibitors promoted maintenance of the cells in hPSC colonies even after induction of differentiation. By contrast, cells cultured without the inhibitors dispersed from the colonies, and hPSC colony integrity was diminished (Figure 3C).

Consistent with the cellular behaviors, IWP2 or XAV939 significantly reduced the expression of mesenchymal genes in hPSCs induced to undergo hematopoietic differentiation, as revealed by qRT‐PCR and gene expression profiling analyses (Figure 3D,E). The impact of Wnt signaling inhibition on hPSC differentiation was profound: IWP2 or XAV939 treatment abrogated mesoderm induction and hematopoietic differentiation toward CD43^+^ and CD45^+^ HPCs (Figure 3F,G; and Figure S3B–D, Supporting Information).

To further test the role of Wnt signaling in mesenchymal gene expression and hematopoietic differentiation, we utilized the Wnt pathway activators CHIR99021 and BIO.^[^ ^33^, ^35^ ^]^ CHIR99021 or BIO treatment upregulated expression of mesenchymal genes (*TBX20*, *BMP2*, and *NRP1*) (Figure 3H). Wnt activation also increased generation of CD43^+^ and CD45^+^ HPCs (Figure 3I; and Figure S3E, Supporting Information). Thus, when activated or inhibited, Wnt signaling, profoundly impacts mesenchymal gene expression, mesoderm induction, and hematopoietic differentiation.

Interestingly, in addition to Wnt signaling, TGF‐*β* signaling‐related genes also showed enrichment in mesoderm cells in comparison to pluripotent stem cells (Figure 3A). Because TGF‐*β* signaling has been described as an important regulator of EMT,^[^ ^25^ ^]^ we assessed whether there was a potential link between Wnt and TGF‐*β* signaling during mesoderm induction. To address this, we utilized phosphorylated of SMAD2/3 and the nuclear accumulation of *β*‐catenin as the readouts for TGF‐*β* signaling and Wnt signaling activation, respectively.^[^ ^36^, ^37^ ^]^ Disruption of Wnt signaling with the specific inhibitor IWP2 led to a significant decrease of phosphorylated SMAD2/3 (Figure S3F, Supporting Information). Furthermore, the expression of *LEFTY1*, a downstream target gene of SMAD2/3,^[^ ^38^ ^]^ was also profoundly suppressed by IWP2 treatment (Figure S3G, Supporting Information). Treatment of a TGF‐*β* signaling inhibitor, Repsox, caused the decrease of nuclear *β*‐catenin in differentiated hESCs (Figure S3H, Supporting Information), while Repsox treatments also markedly suppressed the expression of *AXIN2*, a downstream target gene of Wnt signaling^[^ ^37^ ^]^ (Figure S3I, Supporting Information). These results suggest that there is a crosstalk between Wnt and TGF‐*β* signaling during the early window of hematopoietic differentiation of hPSCs.

### 2.4 Identification of Potential Cell Fate‐Controlling Mesenchymal Genes

**Figure 4:**
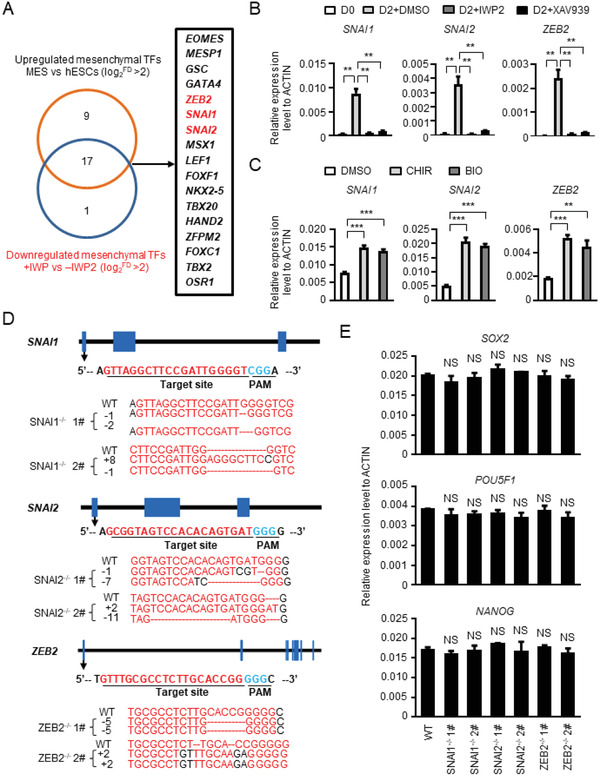
Identification of potential cell fate‐controlling mesenchymal genes. A) The screening strategy for identifying potential cell fate‐controlling mesenchymal genes. B) The expression of *SNAI1, SNAI2*, and *ZEB2* with or without Wnt signaling inhibitor treatment detected with real‐time PCR. C) The expression of *SNAI1, SNAI2, and ZEB2* with or without Wnt signaling activator treatment detected with real‐time PCR. D) Schematic overview of the sgRNA design and the sequence of the targeted sites of *SNAI1* (top), *SNAI2* (middle), and *ZEB2* (bottom). The sgRNA sequences were labeled in red, and the PAM sequences were labeled in blue. E) The expression of representative pluripotency‐maintenance genes *SOX2*, *POU5F1*, and *NANOG* in undifferentiated WT and SNAI1^−/−^, SNAI2^−/−^, ZEB2^−/−^ hESCs analyzed with real‐time PCR. NS, not significant, **P < 0.01 and ***P < 0.001.

After revealing the role of Wnt signaling in the pluripotency to mesoderm transition and concomitant changes in mesenchymal gene expression when Wnt signaling activity was inhibited, we asked whether there were transcription factors (TFs) among the mesenchymal genes that may serve as critical regulators of the fate switch. We enriched significantly upregulated (fold>4) TFs during pluripotency to mesoderm transition, revealing 26 TFs (**Figure** **4**A; and Figure S4A, Supporting Information). We screened significantly downregulated TFs after IWP2 treatment, revealing 18 (Figure S4B, Supporting Information). We found 17 overlapping TFs between the two sets of analyses (Figure 4A), among which several (*EOMES*, *MESP1*, *GATA4*, and *GSC*) have well‐established functions in hPSC early differentiation,^[^ ^39^, ^40^, ^41^, ^42^ ^]^ thus validating our strategy.

*SNAI1*, *SNAI2*, and *ZEB2*, three core EMT transcriptional factors (EMT–TFs),^[^ ^43^ ^]^ were subjected to functional analyses (Figure 4A). As expected, their expression was elevated when Wnt signaling was activated in hPSCs undergoing hematopoietic differentiation and attenuated when Wnt signaling was inhibited (Figure 4B,C). We established knockout cell lines using the CRISPR/Cas9 technology as described.^[^ ^26^ ^]^ Two knock‐out cell lines for *SNAI*, *SNAI2*, and *ZEB2* were established, and the deletion‐ or insertion‐frameshift was confirmed by sequencing (Figure 4D). These gene knockout cell lines can be cultured under pluripotency‐maintaining conditions with minimal impact on pluripotency marker expression (Figure 4E; and Figure S4C, Supporting Information) and are therefore suited for dissecting hPSC differentiation mechanisms.

### 2.5 SNAI1 is Critical for Mesoderm Induction and HPC Generation

**Figure 5:**
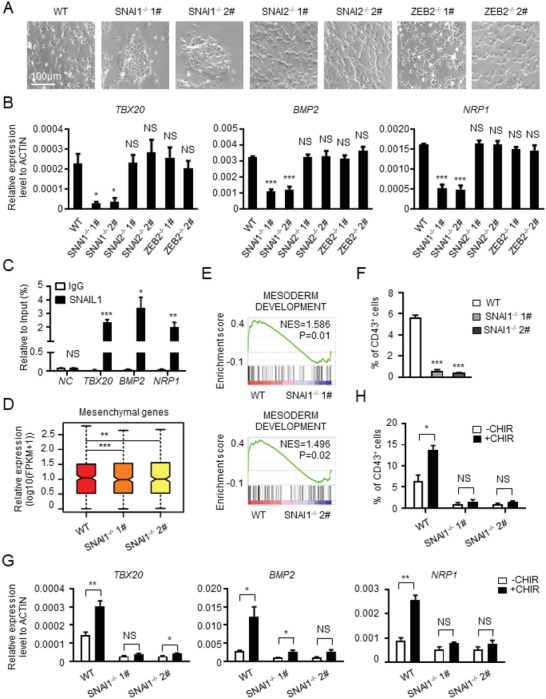
SNAI1 is critical for mesoderm induction and HPC generation. A) The morphology of cells derived from WT and SNAI1^−/−^, SNAI2^−/−^, ZEB2^−/−^ hESCs at day 2 of hematopoietic differentiation. B) The expression of representative mesenchymal genes in the cells derived from WT and SNAI1^−/−^, SNAI2^−/−^, ZEB2^−/−^ hESCs at day 2 of hematopoietic differentiation. C) ChIP‐qPCR analysis of the occupancy of SNAIL1 at the promoter region of *TBX20*, *BMP2*, and *NRP1*. D) The box plot showing the expression of mesenchymal genes in the cells derived from WT and SNAI1^−/−^ hESCs at day 2 of hematopoietic differentiation. E) Comparison of “mesoderm development”‐associated gene expression between the cells differentiated from WT and SNAI1^−/−^ hESCs at day 2 of hematopoietic differentiation. F) The percentage of CD43^+^ HPCs derived from WT and SNAI1^−/−^ hESCs. G) The relative mRNA expression level of representative mesenchymal genes in the cells derived from WT and SNAI1^−/−^ hESCs with or without CHIR treatment. H) The percentage of CD43^+^ HPCs derived from WT and SNAI1^−/−^ hESCs with or without CHIR treatment. NS, not significant, *P < 0.05, **P < 0.01, and ***P < 0.001.

To reveal the critical mesenchymal gene for mesoderm induction and HPC generation, we tested the function of the three TFs. Deletion of SNAI1, but not SNAI2 or ZEB2, prevented hPSCs from acquiring morphologies indicative of differentiation and promoted maintenance of cells in intact colonies, which contrasted with wild‐type cells induced to undergo differentiation (**Figure** **5**A). Consistent with these morphological responses, the deletion of SNAI1, but not SNAI2 or ZEB2, inhibited *TBX20*, *BMP2*, and *NRP1* upregulation (Figure 5B). Chromatin immunoprecipitation‐quantitative polymerase chain reaction (ChIP‐qPCR) analysis revealed that SNAIL1 could bind directly to the promoter of these mesenchymal genes (Figure 5C). RNA‐seq analysis showed that SNAI1 deletion reduced the expression of mesenchymal genes (Figure 5D). Furthermore, SNAI1 deletion reduced the number of APLNR^+^ mesoderm cells. By contrast, SNAI2 or ZEB2 deletion did not affect APLNR^+^ cell generation (Figure 5E; and Figure S5A, Supporting Information).

We also explored the difference in temporal expression between these three transcription factors. Time‐course analysis of gene expression revealed a dramatic upregulation of *SNAI1* within 3 h of hematopoietic differentiation, clearly preceding and higher than *SNAI2* and *ZEB2* expression (Figure S5B, Supporting Information). In addition, SNAI1 deletion significantly repressed the expression of *SNAI2* and *ZEB2* (Figure S5C, Supporting Information). Subsequent ChIP‐qPCR analysis showed that SNAIL1 bound directly to the promoter of *SNAI2* and *ZEB2* (Figure S5D, Supporting Information). In contrast, SNAI2 deletion did not alter the expression of *SNAI1* and *ZEB2*, while ZEB2 deletion also had minimal effects on the expression of *SNAI1* and *SNAI2* (Figure S5E,F, Supporting Information). Thus, SNAI1 deletion suppresses mesenchymal gene expression and impairs mesoderm induction. Thus, SNAI1 acts as the most pivotal gene, and there are no redundant functions among the three genes during the hematopoietic differentiation. In addition to mesoderm induction, we analyzed endothelial and hematopoietic differentiation of SNAI1‐deleted cells. Consistent with the inhibition of mesoderm induction, SNAI1 deletion reduced the generation of CD31^+^CD34^+^ HEPs and CD43^+^ HPCs (Figure 5F; and Figure S5G,H, Supporting Information).

We found earlier that Wnt signaling activation was essential for mesenchymal gene expression, mesoderm induction, and HPC differentiation (Figure 3E–G; and Figure S3C, Supporting Information). Because cells with SNAI1 deletion phenocopied those in which Wnt signaling was inhibited, we tested whether SNAI1 was an effector of Wnt signaling. By using CHIR99021 to activate Wnt signaling in SNAI1‐deleted cells, we quantified the impact on mesenchymal gene expression and cellular differentiation. We found that SNAI1 deletion markedly abrogated CHIR99021‐dependent upregulation of *TBX20*, *BMP2*, and *NRP1* (Figure 5G). No difference was detected in the subsequent generation of CD43^+^ cells in SNAI1‐deleted cells with or without CHIR99021 treatment (Figure 5H). Thus, the genomic and functional analyses identified SNAI1 as a mesenchymal gene essential for the pluripotency to mesoderm transition. Furthermore, SNAIL1 is a key downstream effector that mediates the function of Wnt signaling during this process.

### 2.6 The Suppressive Role of TGF‐*β* Signaling in Downregulation of Mesenchymal Genes and EHT

**Figure 6:**
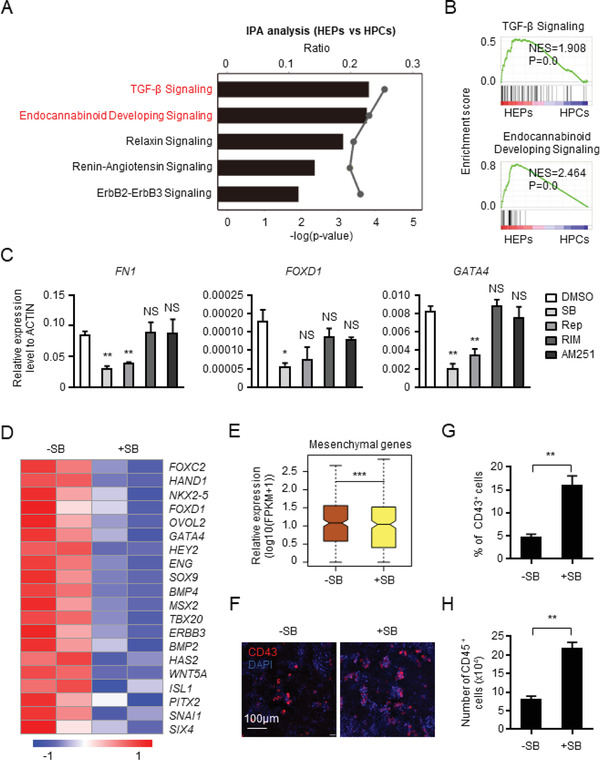
The suppressive role of TGF‐*β* signaling in downregulation of mesenchymal genes and EHT. A) IPA showing the potential signaling pathways controlling transition from HEPs to HPCs. B) Comparison of “TGF‐*β* signaling” and “Endocannabinoid developing signaling”‐associated gene expression between HEPs and HPCs. C) The relative mRNA expression level of representative mesenchymal genes with or without the treatment of SB431542 (SB) and Repsox (Rep), the chemical inhibitors of TGF‐*β* signaling, or Rimonabant (RIM) and AM251, inhibitors of endocannabinoid developing signaling. D) The heatmap showing the expression of mesenchymal genes in the CD31^+^ cells with or without SB treatment. E) The box plot showing the expression of mesenchymal genes in the CD31^+^ cells with or without SB treatment. F) The immunofluorescence of CD43^+^ HPCs with or without SB treatment. G,H) The percentage of CD43^+^ HPCs (G) and the number of CD45^+^ HPCs (H) with or without SB treatment. NS, not significant, *P < 0.05, **P < 0.01, and ***P < 0.001.

The expression pattern of mesenchymal genes was transient during hPSC hematopoietic differentiation; it was elevated during the pluripotency to mesoderm transition and attenuated upon HEP differentiation to HPCs. Because Wnt signaling and its downstream effector SNAI1 were essential for the upregulation of the mesenchymal gene (Figures 3D,E and 5B–D), we tested how mesenchymal genes were downregulated during the HEP–HPC switch and whether their downregulation was essential for the endothelial to EHT. We conducted IPA with RNA‐seq data from cells undergoing EHT, which revealed that TGF‐*β* signaling and endocannabinoid developing signaling are the top enriched responses (**Figure** **6**A). GSEA also demonstrated that the difference in gene expression associated with TGF‐*β* and endocannabinoid developing signaling is statistically significant between HEPs and HPCs (Figure 6B). The gene expression difference occurred in vivo (Figure S6A,B, Supporting Information).

To determine whether TGF‐*β* signaling and/or endocannabinoid signaling were functional, we assessed whether TGF‐*β* signaling inhibitors SB431542 and Repsox^[^ ^22^, ^44^ ^]^ or endocannabinoid signaling inhibitors (Rimonabant and AM251) impacted mesenchymal gene expression and EHT^[^ ^45^, ^46^ ^]^ (Figure S6C, Supporting Information). TGF‐*β* signaling inhibitors suppressed *FN1*, *FOXD1*, and *GATA4* expression (Figure 6C), while inhibition of endocannabinoid developing signaling had no effect (Figure 6C). We conducted RNA‐seq analysis to determine the impact of TGF‐*β* signaling inhibition and found that SB431542 reduced the expression of mesenchymal genes (Figure 6D,E) and elevated CD43^+^ and CD45^+^ HPC generation (Figure 6F–H; and Figure S6D, Supporting Information). Inhibition of endocannabinoid signaling did not affect CD43^+^ cell generation (Figure S6E, Supporting Information). Thus, inhibition of TGF‐*β* signaling augments the downregulation of mesenchymal genes and therefore facilitates hematopoietic differentiation from endothelial cells.

### 2.7 HAND1 Mediates the Suppressive Function of TGF‐*β* Signaling in EHT

**Figure 7:**
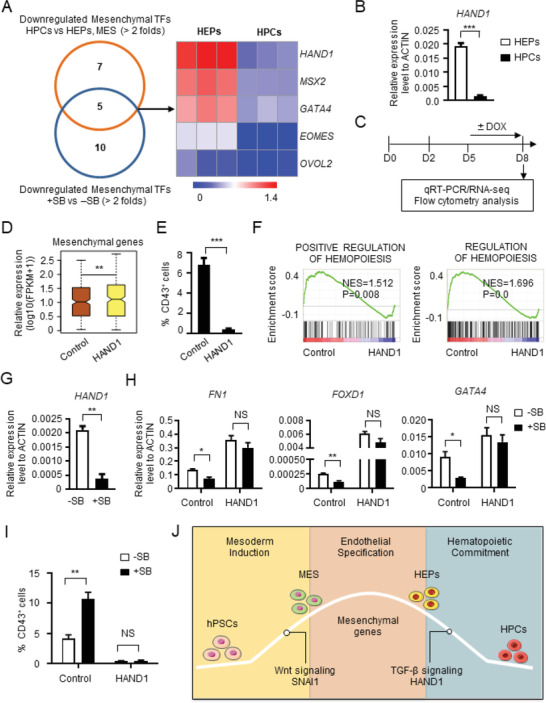
HAND1 mediates the suppressive function of TGF‐*β* signaling in EHT. A) The screening strategy for identifying potential mesenchymal genes regulating the transition from HEPs to HPCs. B) The relative expression level of *HAND1* between HEPs and HPCs. C) The strategy of assessing the expression of mesenchymal genes and the hematopoietic potential with or without HAND1 overexpression. D) The box plot showing the expression level of mesenchymal genes in the CD31^+^ cells with or without *HAND1* overexpression. E) The percentage of CD43^+^ HPCs with or without HAND1 overexpression detected by flow cytometry. F) Comparison of “Positive regulation of hemotopoiesis” and “Regulation of hemotopoiesis” in the CD31^+^ cells with or without HAND1 overexpression. G) The relative expression level of *HAND1* in the CD31^+^ cells with or without SB431542 (SB) treatment detected with real‐time PCR. H) The relative expression level of representative mesenchymal genes in the CD31^+^ cells derived from Control and HAND1 overexpressed hESCs with or without SB treatment detected with real‐time PCR. I) The percentage of CD43^+^ HPCs derived from Control and HAND1 overexpressed hESCs with or without SB treatment analyzed with flow cytometry. J) A working model for the expression pattern, regulation and function of mesenchymal genes during hematopoietic differentiation of hPSCs. NS, not significant, *P < 0.05, **P < 0.01 and ***P < 0.001.

How does TGF‐*β* signaling regulate mesenchymal gene expression and EHT? We applied a similar screening strategy as described earlier for the identification of SNAI1. We overlapped the significantly downregulated TFs (fold>2) during MES and HEP to HPC transition with TFs downregulated upon SB431542 treatment, which revealed five candidate TFs (*HAND1*, *MSX2*, *GATA4*, *EOMES*, and *OVOL2*) (**Figure** **7**A). Among those, a rapid and substantial decrease in expression was confirmed for *HAND1*, *MSX2*, and *GATA4* during the HEP to HPC transition (Figure 7B; and Figure S7A, Supporting Information).

Because HAND1 ranked at the top of the list, we tested whether it is uniquely important. We ectopically expressed HAND1 prior to the HEP to HPC transition and tested its impact on mesenchymal gene expression and hematopoietic differentiation (Figure 7C). This analysis revealed that HAND1 overexpression sufficed to elevate the expression of mesenchymal genes (*FN1*, *FOXD1*, and *GATA4*) (Figure 7D; and Figure S7B, Supporting Information), and there was a direct binding of HAND1 to the promoter region of these mesenchymal genes (Figure S7C, Supporting Information). Consistently, CD43^+^ HPC generation was significantly reduced after HAND1 overexpression during the EHT process (Figure 7E; and Figure S7D, Supporting Information). Experiments with RNA profiling confirmed the expression of hematopoiesis‐associated genes was severely reduced upon HAND1 overexpression (Figure 7F; and Figure S7E, Supporting Information). Thus, HAND1 functions as an essential mesenchymal gene that needs to be downregulated during the HEP to HPC switch. Sustained expression of HAND1 prevented HPC generation from HEPs.

We next tested whether there is a link between TGF‐*β* signaling and HAND1 function. After TGF‐*β* signaling was inhibited with SB431542, HAND1 was significantly downregulated (Figure 7G). By contrast, after HAND1 overexpression, SB431542 failed to suppress the expression of *FN1*, *FOXD1*, and *GATA4* (Figure 7H). When HAND1 was overexpressed, inhibition of TGF‐*β* signaling no longer augmented CD43^+^ HPC generation in comparison with wild‐type cells without HAND1 overexpression (Figure 7I). Thus, HAND1 functions as a downstream signaling effector of TGF‐*β* signaling to prevent the downregulation of mesenchymal genes and EHT.

## 3 Discussion

In this study, we described a biphasic expression pattern of mesenchymal genes during human hematopoiesis from hPSCs. The expression of mesenchymal genes is upregulated from pluripotency to mesoderm, sustained at the HE stages, and attenuated during HEP differentiation to HPCs. A similar expression pattern of mesenchymal genes was also observed during human and murine early hematopoietic development in vivo. Mechanistically, Wnt signaling triggers expression of SNAI1 and other mesenchymal genes and induces differentiation of pluripotent stem cells definition throughout to mesoderm and subsequently to HPCs (Figure 7J). Moreover, suppression of mesenchymal gene expression is required for the successful fate switch from HEPs to HPCs, and this is accomplished via inhibition of TGF‐*β* signaling and downregulation of HAND1, a mesenchymal gene downstream of TGF‐*β* (Figure 7J). Our results revealed new insights into the mechanisms governing human hematopoiesis and may, therefore, facilitate the establishment of novel strategies for the efficient production of functional blood cells from hPSCs for regenerative medicine.

A sequential EMT–MET process occurs and plays a critical role during the processes of embryonic development, tumorigenesis, cellular programming, and reprogramming.^[^ ^4^, ^5^, ^47^ ^]^ However, whether a similar mechanism underlines hematopoietic development remains unknown. Here, we demonstrated that typical EMT exists during the fate switch from pluripotency to mesoderm during human hematopoiesis. However, in the subsequent differentiation stages (i.e., from mesoderm to HEPs and HEPs to HPCs), MET is not detected, unlike many other developmental and disease models, such as the differentiation of hESCs toward hepatocytes, a type of epithelial cells.^[^ ^44^ ^]^ In contrast, biphasic activation of mesenchymal genes was also observed during early hematopoietic development in vitro and in vivo. Thus, these results imply that biphasic regulation of mesenchymal gene expression, rather than a sequential EMT–MET, is essential for the fate switches during human hematopoiesis. To our knowledge, this is the first comprehensive study that elucidates the role of EMT–MET in human hematopoiesis at the transcriptome level. These findings provide novel and important insights for the understanding of the dynamic changes of mesenchymal genes during hematopoietic fate decisions. Notably, we found that some mesenchymal genes, such as *TWIST1* and *ZEB2*, remain highly expressed at the HPC stage, consistent with their established critical roles in the generation and differentiation of embryonic hematopoietic stem/progenitor cells.^[^ ^11^, ^14^, ^15^ ^]^

We found that inactivation of Wnt signaling or deletion of SNAI1 in hPSCs severely impairs the derivation of mesoderm cells, consistent with prior observations.^[^ ^19^, ^20^, ^21^, ^48^, ^49^ ^]^ However, little is known about whether and how Wnt signaling or SNAI1 regulates the expression of other mesenchymal genes. We demonstrated that inactivation of Wnt signaling attenuates the expression of a large group of mesenchymal genes. Thus, it is tempting to propose that regulation of mesenchymal genes expression serves as one of the essential functions of Wnt signaling. In this study, we find that three core EMT–TFs, SNAI1, SNAI2, and ZEB2, are upregulated during mesoderm induction and regulated by Wnt signaling. There are no redundant functions among these EMT–TFs during hematopoietic differentiation. This observation is consistent with distinct and nonredundant functions of core EMT–TFs in a context‐dependent manner.^[^ ^43^ ^]^ For example, knockout of SNAI1, but not SNAI2, ZEB1, and ZEB2, impairs the differentiation of hESCs toward hepatocytes.^[^ ^44^ ^]^ In addition, we present evidence that SNAIL1 controls the expression of multiple mesenchymal genes, including the other core EMT–TFs, by directly binding to the promoter region of these genes, implying that SNAI1 acts at a higher position of the signaling hierarchy and might mediate the expression of other mesenchymal genes during the hematopoietic differentiation of hPSCs.

EHT serves as a vital process for the initiation of hematopoiesis and is under precise regulation. In this study, we discovered that the downregulation of mesenchymal genes induced by inhibition of TGF‐*β* signaling is critical for the HEP to HPC transition. Although the suppressive role of TGF‐*β* signaling in EHT has been documented,^[^ ^22^, ^23^, ^24^ ^]^ little is known about how TGF‐*β* signaling exerts its function. We found that TGF‐*β* regulation of the expression of mesenchymal genes plays a pivotal role in this process. Furthermore, we identified HAND1 as a key mesenchymal gene mediating the suppressive role of TGF‐*β* signaling, and its downregulation facilitates HPC generation from HEPs. Our findings unveil a novel function of HAND1 in EHT and establish a link between HAND1 and TGF‐*β* signaling during human early hematopoietic development. A more comprehensive understanding of the detailed mechanisms underlying mesenchymal gene regulation by upstream signaling pathways and mutual regulation among mesenchymal genes during early human hematopoiesis awaits future experimentation.

## 4 Conclusion

In summary, we observe, for the first time, biphasic activation of mesenchymal genes during hematopoietic differentiation of hPSCs. This pattern occurs during human and murine hematopoietic development in vivo. At the mechanistic level, Wnt signaling and its downstream SNAI1 mediate the up‐regulation of mesenchymal genes and initiation of mesoderm induction from pluripotency, while inhibition of TGF‐*β* signaling and downregulation of HAND1 are required for the generation of HPCs from HEPs. Our discoveries revealed new insights into the molecular basis underlying early human hematopoietic development and should benefit the production of HSCs and functional blood cells from hPSCs for clinical applications.

## 5 Experimental Section

##### Cell Line and Cell Culture

H1 hESCs (WiCell Research Institute, Madison, WI) and BC1 hiPSCs (kindly provided by Linzhao Cheng, Johns Hopkins University) were maintained on Matrigel‐coated 12‐well‐plate and fresh mTeSR medium was changed every day. Every 3–4 days, cells were dissociated by Dispase (2 U mL^−1^) and passaged at the dilution of 1:4.

##### Hematopoietic Differentiation

H1 and BC1 were induced into hematopoietic differentiation using the chemical defined system. And Custom TeSR supplemented with 1% penicillin/streptomycin and 0.05% plasmocin were used as hematopoietic differentiation medium. For the start, cells were digested by Accutase and plated on Growth Factor Reduced Matrigel‐coated 12‐well‐plate at the density of 3–3.5 × 10^4^ cells per well. The next day, bone morphogenetic protein 4 (BMP4) (40 ng mL^−1^) and Activin A (50 ng mL^−1^) were added into the medium to induce mesoderm commitment. 48 h later, cells were fed with medium supplemented with vascular endothelial growth factor (40 ng mL^−1^) and basic fibroblast growth factor (50 ng mL^−1^), and the medium was changed every day for 5–6 days. For the further induction of CD45^+^ hematopoietic cells, differentiated cells at day 7–8 were harvested by Accutase and replated onto low‐attachment 24‐well‐plates at the density of 1 × 10^5^/well and fed with Custom TeSR supplemented with 1% insulin‐transferrin‐selenium, glutamax (1 × 10^−3^ m), 1% NEAA, 2% B27 (all from Gibco), monothioglycerol (0.1 × 10^−3^ m), interleukin‐3 (20 ng mL^−1^), stem cell factor (20 ng mL^−1^), thrombopoietin (50 ng mL^−1^). Fresh medium was changed every 2 days.

##### RNA‐Seq

RNA‐seq analysis was performed by BGI Company (BGI, Shenzhen, China) and Novogene Company (Tianjin, China). R language was used to generate Heatmap, box plot and violin plot. PCA was conducted on ClustVis website (http://biit.cs.ut.ee/clustvis/). GO was performed with the online tool (http://david.abcc.ncifcrf.gov/). IPA software was used for the canonical signaling pathway analysis. Functional gene sets were downloaded from the web (http://software.broadinstitute.org/gsea/index.jsp), and GSEA software was used to conduct enrichment analysis. The RNA‐seq data are available at Gene Expression Omnibus (GEO) website (Accession number: GSE134907, GSE134905, GSE134910, GSE134911, GSE134908, and GSE134906).

##### Single‐Cell RNA Sequencing

The raw data for Single Cell RNA Sequencing (SC RNA‐Seq) was obtained from the previously published manuscript.^[^ ^31^ ^]^ Epiblast, Mesoderm, Endothelium, Blood progenitors, and Embryonic blood were chosen as the target cell populations. Seurat (version 2.3.4)^[^ ^50^ ^]^ and Monocle2 packages^[^ ^51^ ^]^ were used to perform further analysis. Gene counts were log transformed and normalized by “Normalize Data” function from Seurat with default parameters. PCs 1–10 were used to perform dimensional reduction, the identification of cells were defined by previously described in the original article.^[^ ^31^ ^]^ Monocle2 was used to conduct pseudotime analysis with default parameters.

##### Statistical Analysis

At least three independent experiments were performed for each analysis, and the data were shown as the mean ± SD. The differences were analyzed with unpaired Student‐*t* test or paired Student‐*t* test using the GraphPad Prism software. Differences were considered statistically significant when *P* < 0.05. NS, not significant; *P < 0.05; ** *P* < 0.01; ***P < 0.001.

## Conflict of Interest

The authors declare no conflict of interest.

## Author Contributions

H.W., M.W., and Y.W. contributed equally to this work. J.Z., L.S., T.C., and H.W. designed the study; H.W., M.W., Y.W., X.C., and P.S performed the functional experiments; C.X., Y.W., H.W., and D.W. performed the bioinformatics analysis with help from J.Z., L.S., T.C., and W.Z.; J.Z., L.S. and H.W. wrote the manuscript.

## Supporting information

Supporting Information
