# Supplementary material for: Biphasic Regulation of Mesenchymal Genes Controls Fate Switches During Hematopoietic Differentiation of Human Pluripotent Stem Cells
Source: Adv Sci (Weinh). 2020 Aug 19;7(20):2001019. doi: 10.1002/advs.202001019 (PMC7578858; doi:10.1002/advs.202001019)
Supplement: Supplementary file 1 — Supporting Information [file ADVS-7-2001019-s001.pdf]

© 2020 Wiley-VCH GmbH

ADVANCED SCIENCE
Open Access

Supporting Information

for *Adv. Sci.,* DOI: 10.1002/advs.202001019

Biphasic Regulation of Mesenchymal Genes Controls Fate Switches During Hematopoietic Differentiation of Human Pluripotent Stem Cells

Hongtao Wang, Mengge Wang, Yuqi Wen, Changlu Xu, Xiaoyuan Chen, Dan Wu, Pei Su, Wen Zhou, Tao Cheng, Lihong Shi, and Jiaxi Zhou**

Supplemental Information

Biphasic Regulation of Mesenchymal Genes Controls Fate Switches During Hematopoietic Differentiation of Human Pluripotent Stem Cells

Hongtao Wang[1,2§], Mengge Wang[1,2§], Yuqi Wen[1,2§], Changlu Xu[1,2], Xiaoyuan Chen[1,2], Dan Wu[1,2], Pei Su[1,2], Wen Zhou[3], Tao Cheng[1,2], Lihong Shi[1,2]*, Jiaxi Zhou[1,2]*

The Following Files are Included:

Supplemental Figures (Figure S1-S7)

Supplemental Figure Legends

Supplemental Experimental Section

Supplemental Tables (Table S1-S6)

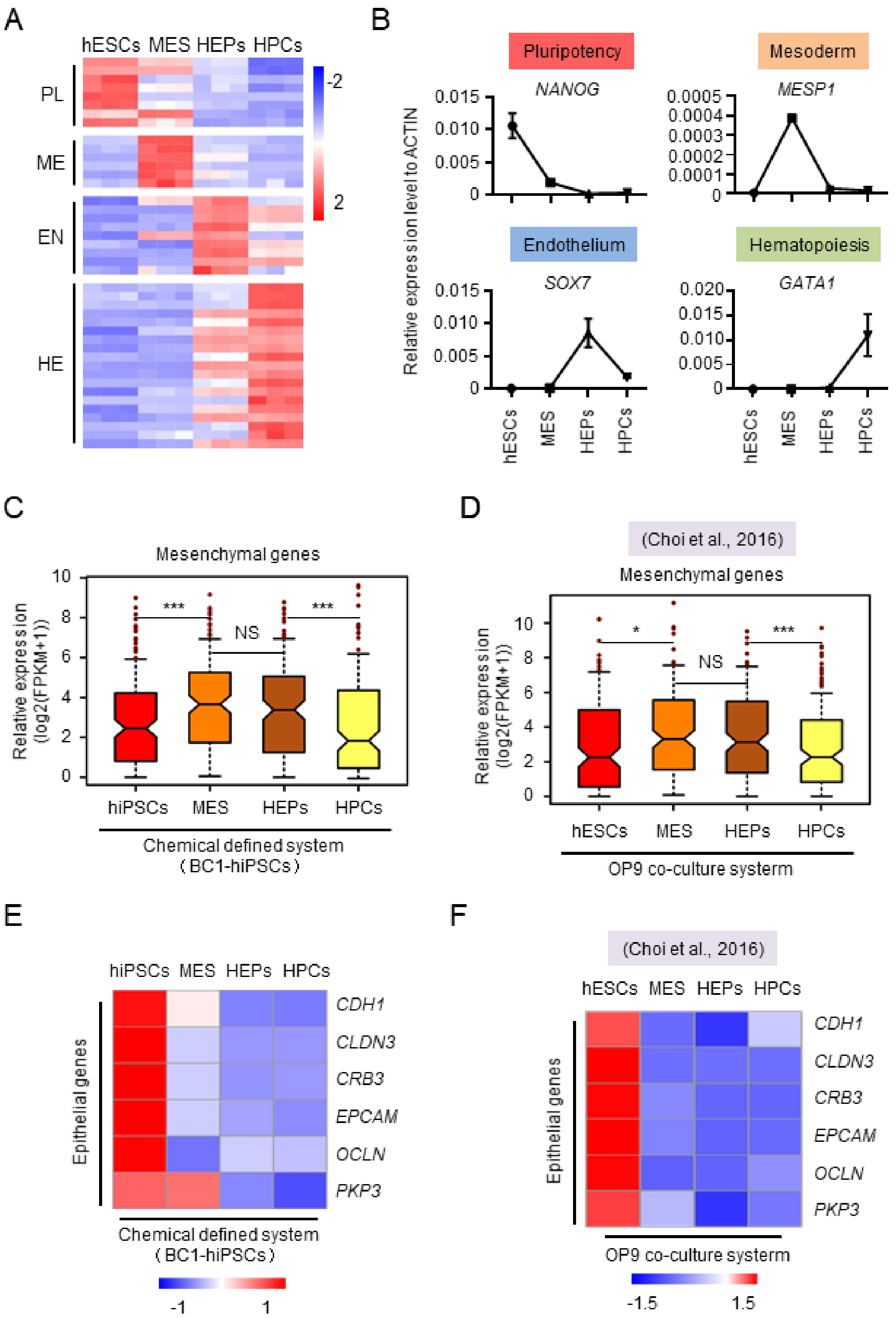
A
hESCs MES HEPs HPCs
PL
ME
EN
HE
-2
2
B
Pluripotency
Mesoderm
Endothelium
Hematopoiesis
NANOG
MESP1
SOX7
GATA1
Relative expression level to ACTIN
hESCs
MES
HEPs
HPCs
C
Mesenchymal genes
Relative expression (log2(FPKM+1))
***
NS
***
hiPSCs MES HEPs HPCs
Chemical defined system (BC1-hiPSCs)
D
(Choi et al., 2016)
Mesenchymal genes
Relative expression (log2(FPKM+1))
*
NS
***
hESCs MES HEPs HPCs
OP9 co-culture systerm
E
hiPSCs MES HEPs HPCs
Epithelial genes
CDH1
CLDN3
CRB3
EPCAM
OCLN
PKP3
Chemical defined system (BC1-hiPSCs)
-1
1
F
(Choi et al., 2016)
hESCs MES HEPs HPCs
Epithelial genes
CDH1
CLDN3
CRB3
EPCAM
OCLN
PKP3
OP9 co-culture systerm
-1.5
1.5

Fig. S1

Figure. S1 Biphasic Regulation of Mesenchymal Genes during Hematopoietic Differentiation of hPSCs. Related to Figure 1

(A) The heatmap of characteristic genes for each population of cells during hematopoietic differentiation of hESCs. (B) The expression of representative genes in four different populations of cells during hematopoietic differentiation of hESCs, as detected with real-time PCR. (C) The box plot showing the different expression patterns of mesenchymal genes between the four populations of cells derived from BC1 hiPSCs during hematopoietic differentiation. (D) The box plot showing the different expression patterns of mesenchymal genes between the four populations of cells derived from hESCs during hematopoietic differentiation in the OP9 co-culture system. (E) The heatmap showing down-regulation of epithelial genes along hematopoietic differentiation of BC1. (F) The heatmap showing down-regulation of epithelial genes during hematopoietic differentiation in the OP9 co-culture system. NS, not significant, *P<0.05 and ***P<0.001.

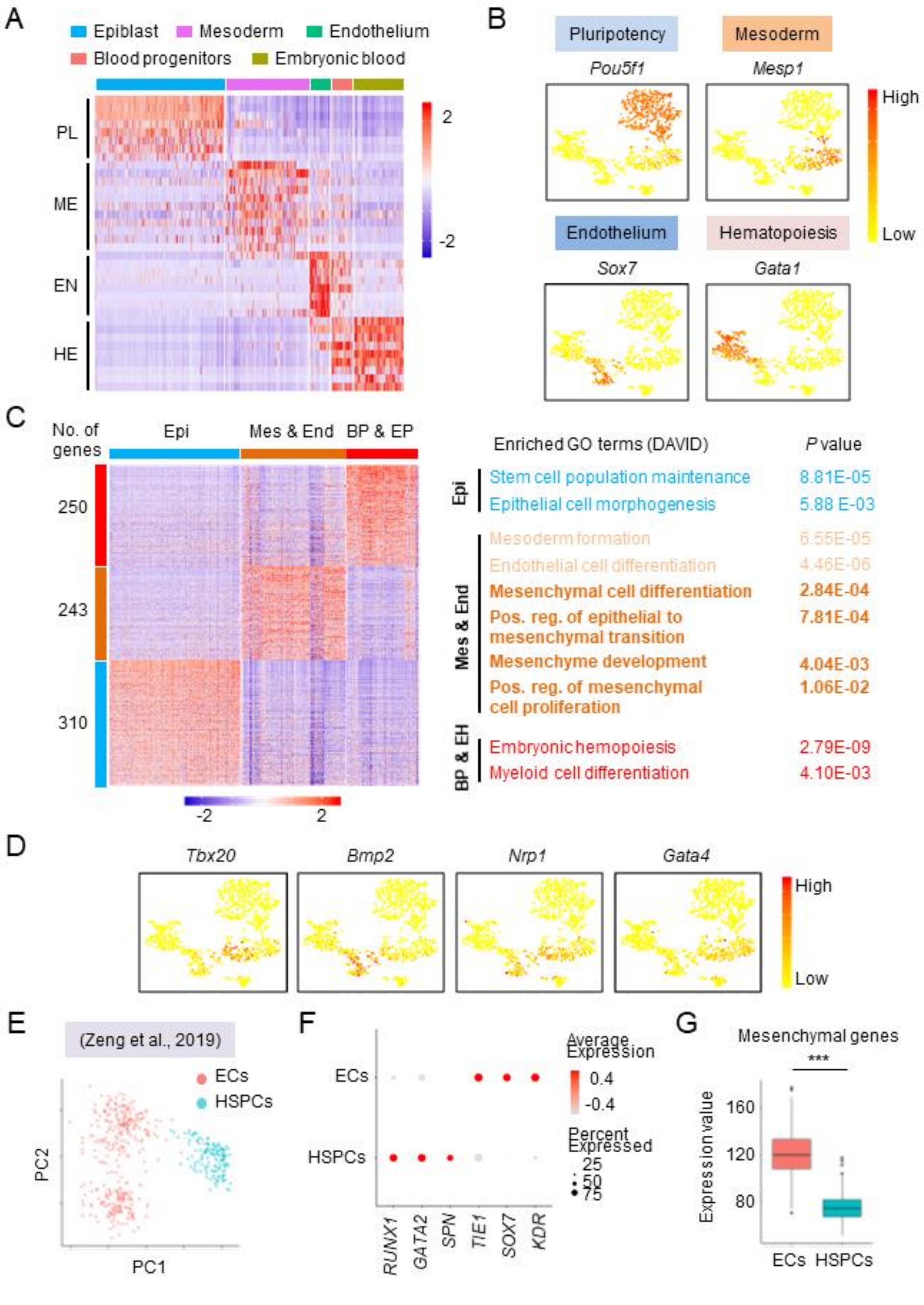
A
Epiblast
Mesoderm
Endothelium
Blood progenitors
Embryonic blood
PL
ME
EN
HE
2
-2
B
Pluripotency
Mesoderm
Pou5f1
Mesp1
High
Low
Endothelium
Hematopoiesis
Sox7
Gata1
C
No. of genes
Epi
Mes & End
BP & EP
250
243
310
-2
2
Enriched GO terms (DAVID)
P value
Epi
Stem cell population maintenance 8.81E-05
Epithelial cell morphogenesis 5.88 E-03
Mes & End
Mesoderm formation 6.55E-05
Endothelial cell differentiation 4.46E-06
Mesenchymal cell differentiation 2.84E-04
Pos. reg. of epithelial to mesenchymal transition 7.81E-04
Mesenchyme development 4.04E-03
Pos. reg. of mesenchymal cell proliferation 1.06E-02
BP & EH
Embryonic hemopoiesis 2.79E-09
Myeloid cell differentiation 4.10E-03
D
Tbx20
Bmp2
Nrp1
Gata4
High
Low
E
(Zeng et al., 2019)
ECs
HSPCs
PC2
PC1
F
Average Expression
0.4
-0.4
Percent Expressed
25
50
75
ECs
HSPCs
RUNX1
GATA2
SPN
TIE1
SOX7
KDR
G
Mesenchymal genes
***
Expression value
160
120
80
ECs
HSPCs

Fig. S2

Figure. S2 Biphasic Regulation of Mesenchymal Genes Occurs during Hematopoietic Development *in vivo*. Related to Figure 2

(A) The heatmap showing the expression of characteristic genes for each population of cells during mouse embryonic hematopoiesis *in vivo*. (B) t-SNE showing the expression of representative genes for each population of cells during mouse embryonic hematopoiesis *in vivo*. (C) The gene sets associated with mesenchymal development were enriched in mesoderm and endothelium cells, as analyzed by GO enrichment analysis. (D) The dynamic expression of representative mesenchymal genes during mouse embryonic hematopoiesis *in vivo*. (E) PCA plot of endothelial cells (ECs) and hematopoietic stem and progenitor cells (HSPCs) during human hematopoietic development (CS12-CS15) *in vivo*. (F) Dot plots showing the scaled expression level of feature genes in the ECs and HSPCs during human hematopoietic development. (G) The box plot showing the expression of mesenchymal genes in ECs and HSPCs during human hematopoietic development. PL, pluripotency, ME, mesoderm, EN, endothelium, HE, hematopoiesis. Epi, Epiblast, Mes, Mesoderm, End, Endothelium, BP, Blood progenitors, EB, embryonic blood. ***P<0.001.

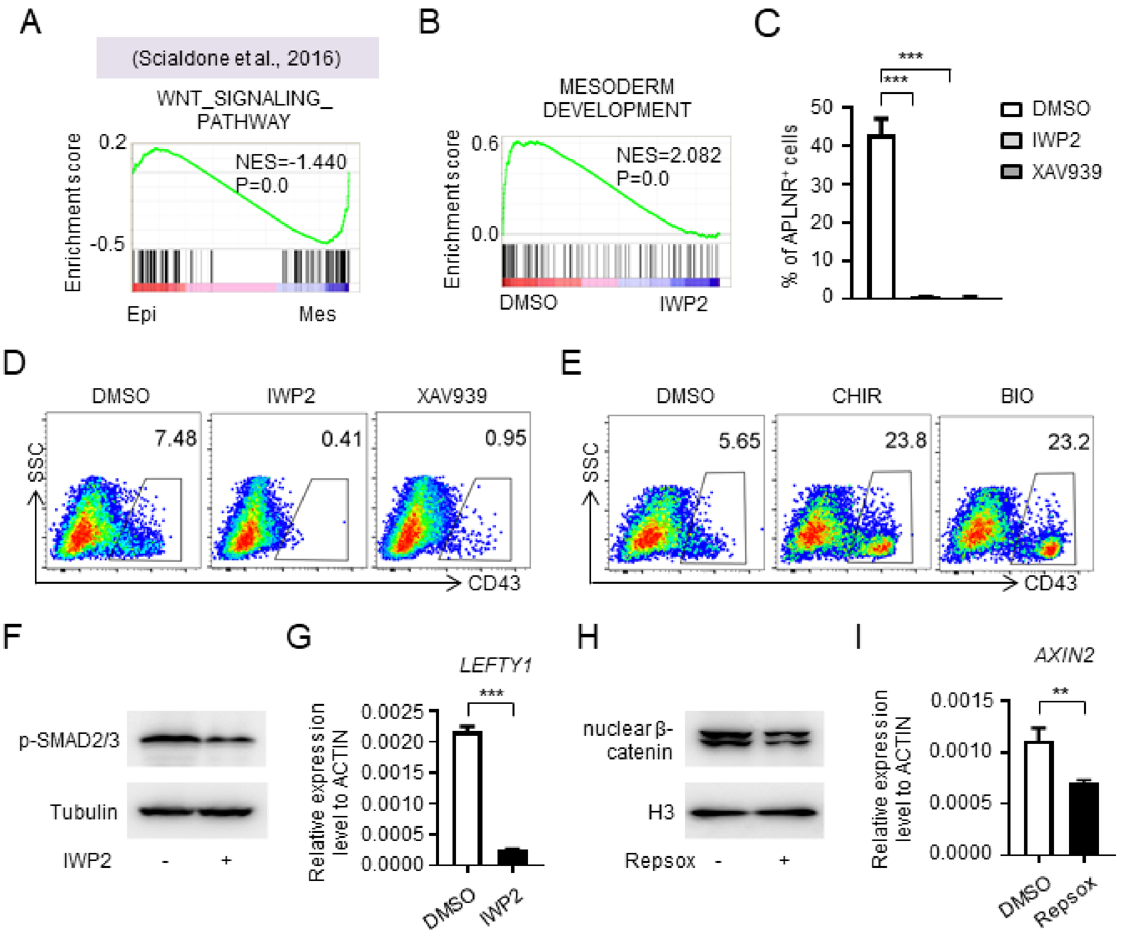
A
(Scialdone et al., 2016)
WNT_SIGNALING_ PATHWAY
Enrichment score
0.2
-0.5
NES=-1.440
P=0.0
Epi
Mes
B
MESODERM DEVELOPMENT
Enrichment score
0.6
0.0
NES=2.082
P=0.0
DMSO
IWP2
C
% of APLNR+ cells
***
***
DMSO
IWP2
XAV939
D
DMSO
IWP2
XAV939
7.48
0.41
0.95
SSC
CD43
E
DMSO
CHIR
BIO
5.65
23.8
23.2
SSC
CD43
F
p-SMAD2/3
Tubulin
IWP2 - +
G
LEFTY1
Relative expression level to ACTIN
***
DMSO
IWP2
H
nuclear β-catenin
H3
Repsox - +
I
AXIN2
Relative expression level to ACTIN
**
DMSO
Repsox

Fig. S3

Figure. S3 Wnt Signaling is Essential for Mesenchymal Gene Expression and Induction of Differentiation. Related to Figure 3

(A) GSEA showing the enrichment of 'Wnt signaling pathway' gene set in mesoderm compared with epiblasts *in vivo*. (B) GSEA showing the enrichment of 'Mesenchyme development' gene set in DMSO-treated cells compared with IWP2-treated cells. (C) The percentage of $APLNR^+$ mesoderm cells with or without Wnt signaling inhibitor treatment at day 2 of hematopoietic differentiation. (D) Flow cytometry analysis of the percentage of $CD43^+$ HPCs with or without the treatments of Wnt signaling inhibitors. (E) Flow cytometry analysis of the percentage of $CD43^+$ HPCs with or without the treatments of Wnt signaling activators. (F) Western blotting of p-SMAD2/3 with or without IWP2 treatment during the stage of mesoderm induction. (G) Real-time PCR analysis of the expression of *LEFTY1* with or without IWP2 treatment during mesoderm induction. (H) Western blotting of nuclear β-catenin with or without Repsox treatment during mesoderm induction. (I) Real-time PCR analysis of the expression of *AXIN2* with or without Repsox treatment during mesoderm induction. $^{**}P<0.01$ and $^{***}P<0.001$.

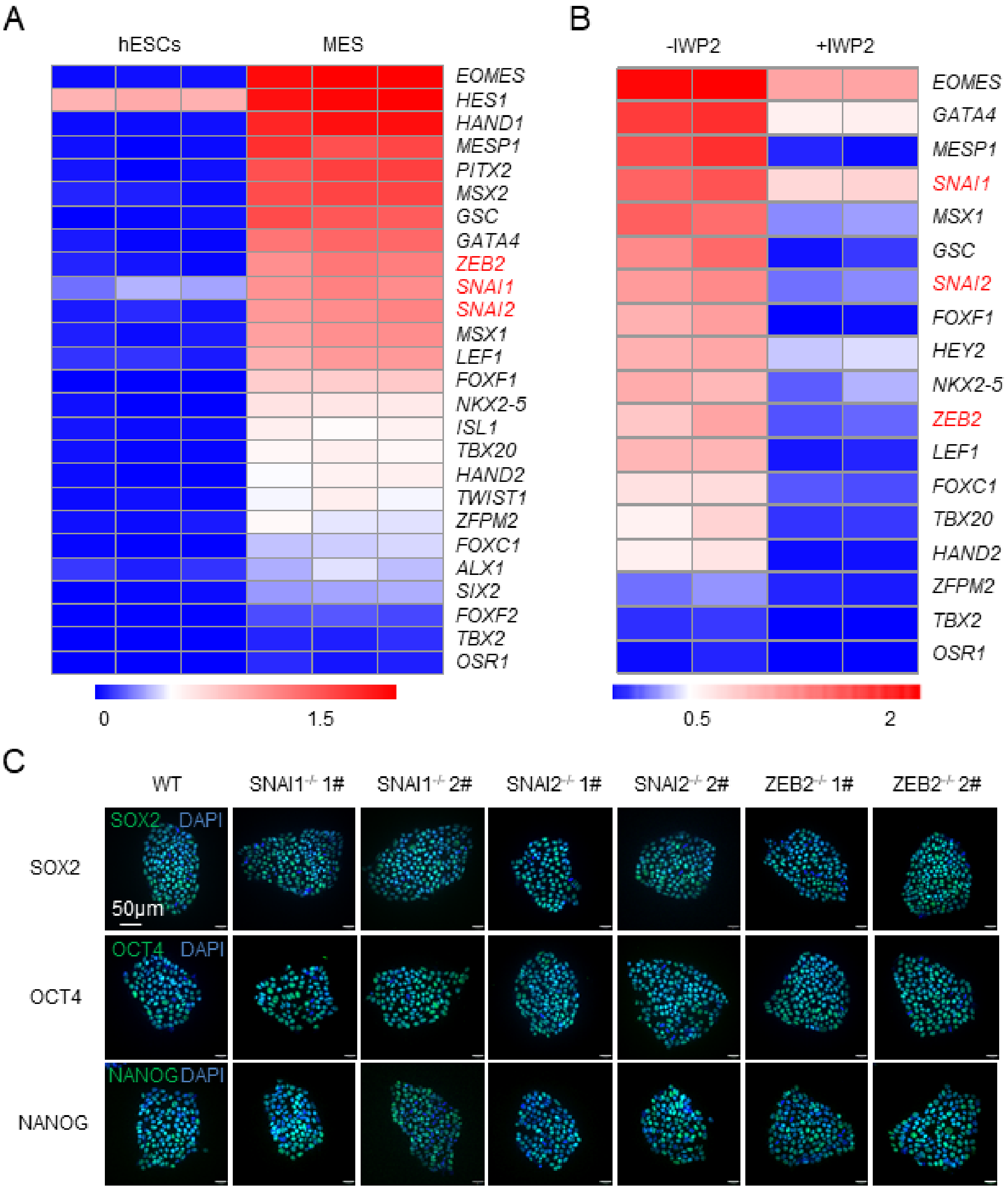
A
hESCs
MES
EOMES
HES1
HAND1
MESP1
PITX2
MSX2
GSC
GATA4
ZEB2
SNAI1
SNAI2
MSX1
LEF1
FOXF1
NKX2-5
ISL1
TBX20
HAND2
TWIST1
ZFPM2
FOXC1
ALX1
SIX2
FOXF2
TBX2
OSR1
0
1.5
B
-IWP2
+IWP2
EOMES
GATA4
MESP1
SNAI1
MSX1
GSC
SNAI2
FOXF1
HEY2
NKX2-5
ZEB2
LEF1
FOXC1
TBX20
HAND2
ZFPM2
TBX2
OSR1
0.5
2
C
WT
SNAI1-/- 1#
SNAI1-/- 2#
SNAI2-/- 1#
SNAI2-/- 2#
ZEB2-/- 1#
ZEB2-/- 2#
SOX2
OCT4
NANOG
SOX2 DAPI
OCT4 DAPI
NANOG DAPI
50μm

Fig. S4

Figure. S4 Identification of Potential Cell Fate-Controlling Mesenchymal Genes. Related to Figure 4

(A) Heatmap showing the expression of 26 mesenchymal genes upregulated in mesoderm cells compared with hESCs. (B) Heatmap showing the expression of 18 mesenchymal genes down-regulated by IWP2 treatment. (C) The immunofluorescence of SOX2, OCT4 and NANOG in undifferentiated WT and $SNAI1^{-/-}$, $SNAI2^{-/-}$, $ZEB2^{-/-}$ hESCs.

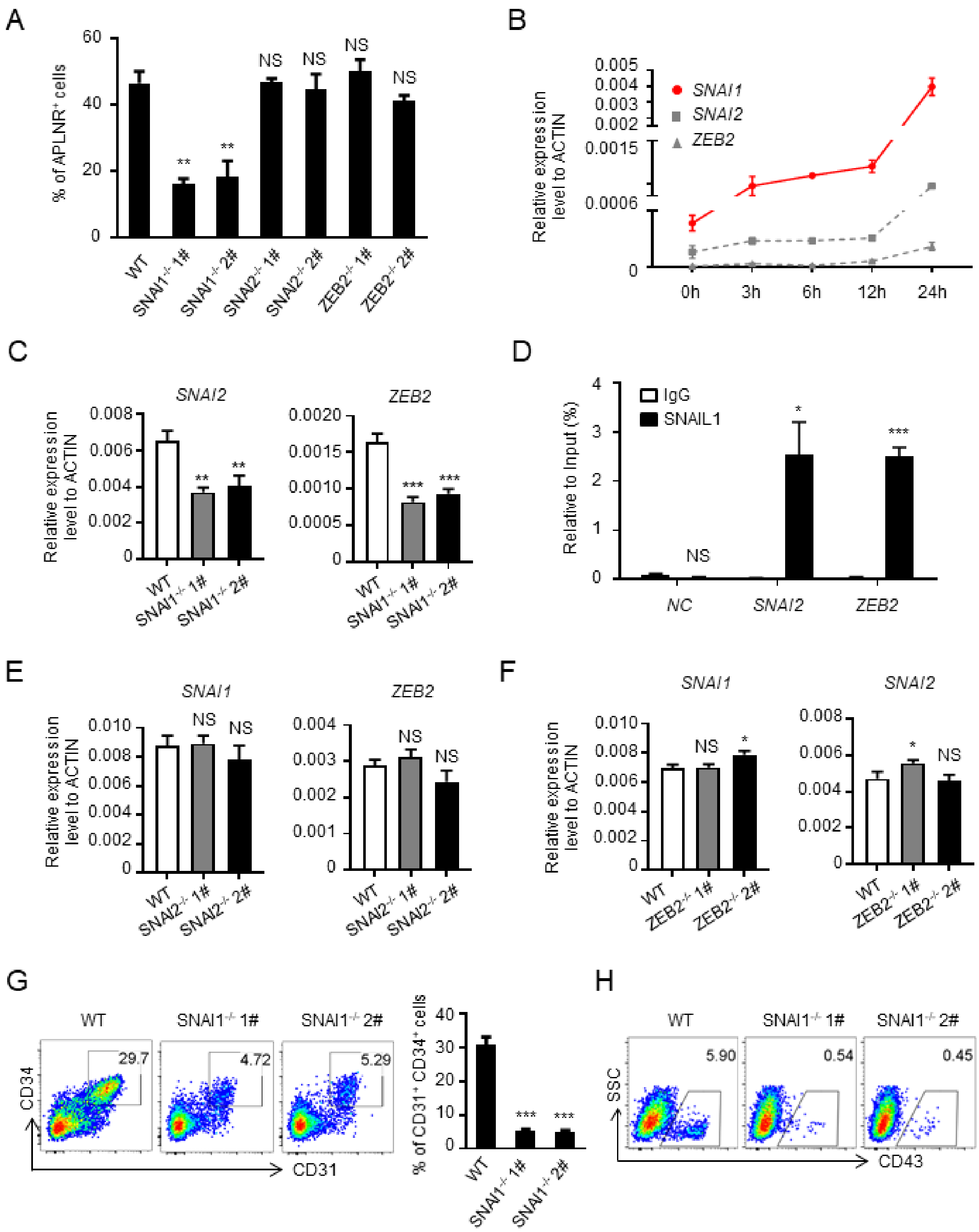
A
% of APLNR⁺ cells
WT
SNAI1-/- 1#
SNAI1-/- 2#
SNAI2-/- 1#
SNAI2-/- 2#
ZEB2-/- 1#
ZEB2-/- 2#
NS
B
Relative expression level to ACTIN
SNAI1
SNAI2
ZEB2
0h
3h
6h
12h
24h
C
SNAI2
ZEB2
Relative expression level to ACTIN
D
IgG
SNAIL1
Relative to Input (%)
NC
SNAI2
ZEB2
NS
E
SNAI1
ZEB2
Relative expression level to ACTIN
F
SNAI1
SNAI2
Relative expression level to ACTIN
G
WT
SNAI1-/- 1#
SNAI1-/- 2#
CD34
CD31
29.7
4.72
5.29
% of CD31⁺ CD34⁺ cells
H
WT
SNAI1-/- 1#
SNAI1-/- 2#
SSC
CD43
5.90
0.54
0.45

Fig. S5

Figure. S5 SNAI1 is Critical for Mesoderm Induction and HPC Generation. Related to Figure 5

(A) The percentage of $APLNR^{+}$ mesoderm cells derived from WT and $SNAI1^{-/-}$, $SNAI2^{-/-}$, $ZEB2^{-/-}$ hESCs at day 2 of hematopoietic differentiation. (B) Real-time PCR analysis of *SNAI1*, *SNAI2* and *ZEB2* expression from 0 h to 24 h after hematopoietic differentiation of hESCs. (C) Real-time PCR analysis of *SNAI2* and *ZEB2* expression in cells derived from WT and $SNAI1^{-/-}$ H1 cells at day 2 of hematopoietic differentiation; (D) ChIP-qPCR analysis of the occupancy of SNAIL1 at the promoter region of *SNAI2* and *ZEB2*. (E) Real-time PCR analysis of the expression of *SNAI1* and *ZEB2* in cells derived from WT and $SNAI2^{-/-}$ H1 cells at day 2 of hematopoietic differentiation; (F) Real-time PCR analysis of the expression of *SNAI1* and *SNAI2* in cells derived from WT and $ZEB2^{-/-}$ H1 cells at day 2 of hematopoietic differentiation; (G) Flow cytometry analysis of the percentage of $CD31^{+}CD34^{+}$ HEPs derived from WT and $SNAI1^{-/-}$ hESCs. (H) Flow cytometry analysis of the percentage of $CD43^{+}$ HPCs derived from WT and $SNAI1^{-/-}$ hESCs. NS, not significant, *$P<0.05$, **$P<0.01$ and ***$P<0.001$.

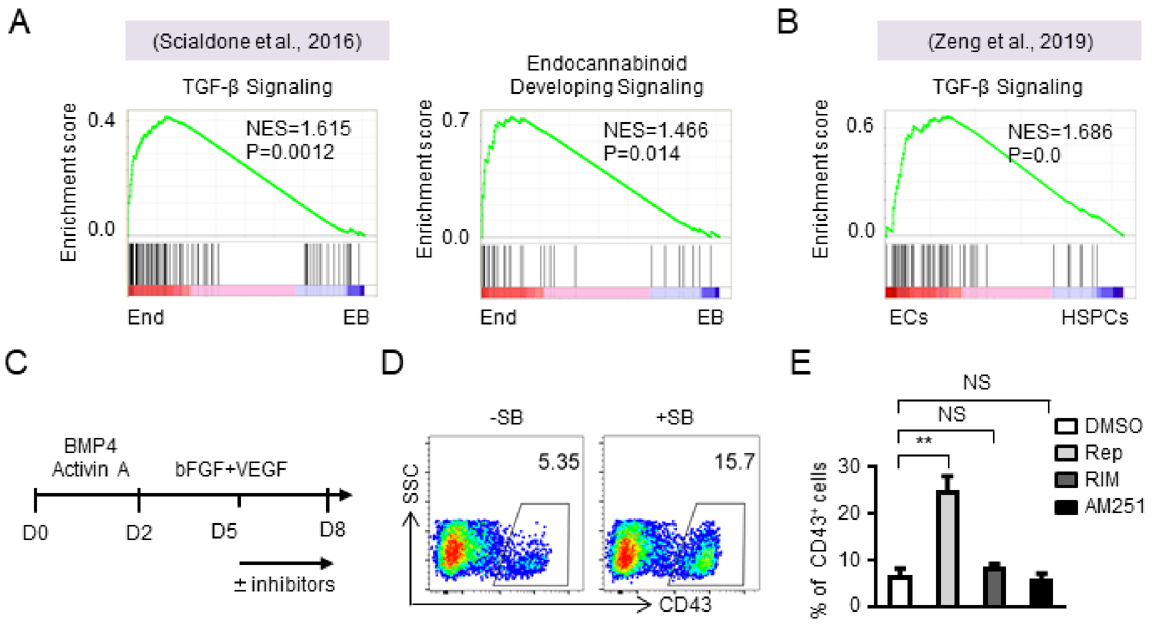
A
(Scialdone et al., 2016)
TGF-β Signaling
Enrichment score
0.4
0.0
NES=1.615
P=0.0012
End
EB
Endocannabinoid
Developing Signaling
0.7
0.0
NES=1.466
P=0.014
End
EB
B
(Zeng et al., 2019)
TGF-β Signaling
0.6
0.0
NES=1.686
P=0.0
ECs
HSPCs
C
BMP4
Activin A
bFGF+VEGF
D0
D2
D5
D8
± inhibitors
D
-SB
+SB
5.35
15.7
SSC
CD43
E
NS
NS
**
DMSO
Rep
RIM
AM251
% of CD43+ cells
30
20
10
0

Fig.S6

Figure. S6 The Suppressive Role of TGF-β Signaling in Downregulation of Mesenchymal Genes and EHT. Related to Figure 6

(A) Comparison of 'TGF-β signaling' and 'endocannabinoid developing signaling' gene sets between endothelium (End) and embryonic blood (EB) during mouse embryonic hematopoiesis *in vivo*. (B) Comparison of 'TGF-β signaling'-associated gene expression between endothelial cells (ECs) and hematopoietic stem and progenitor cells (HSPCs) during human hematopoietic development. (C) The strategy of detecting the signaling inhibitors on the expression of mesenchymal genes and the production of hematopoietic cells. (D) Flow cytometry analysis of $CD43^+$ HPCs with or without SB431542 treatment. (E) The percentage of $CD43^+$ HPCs with or without the inhibitors of TGF-β signaling and endocannabinoid developing signaling treatment. NS, not significant, **$P<0.01$.

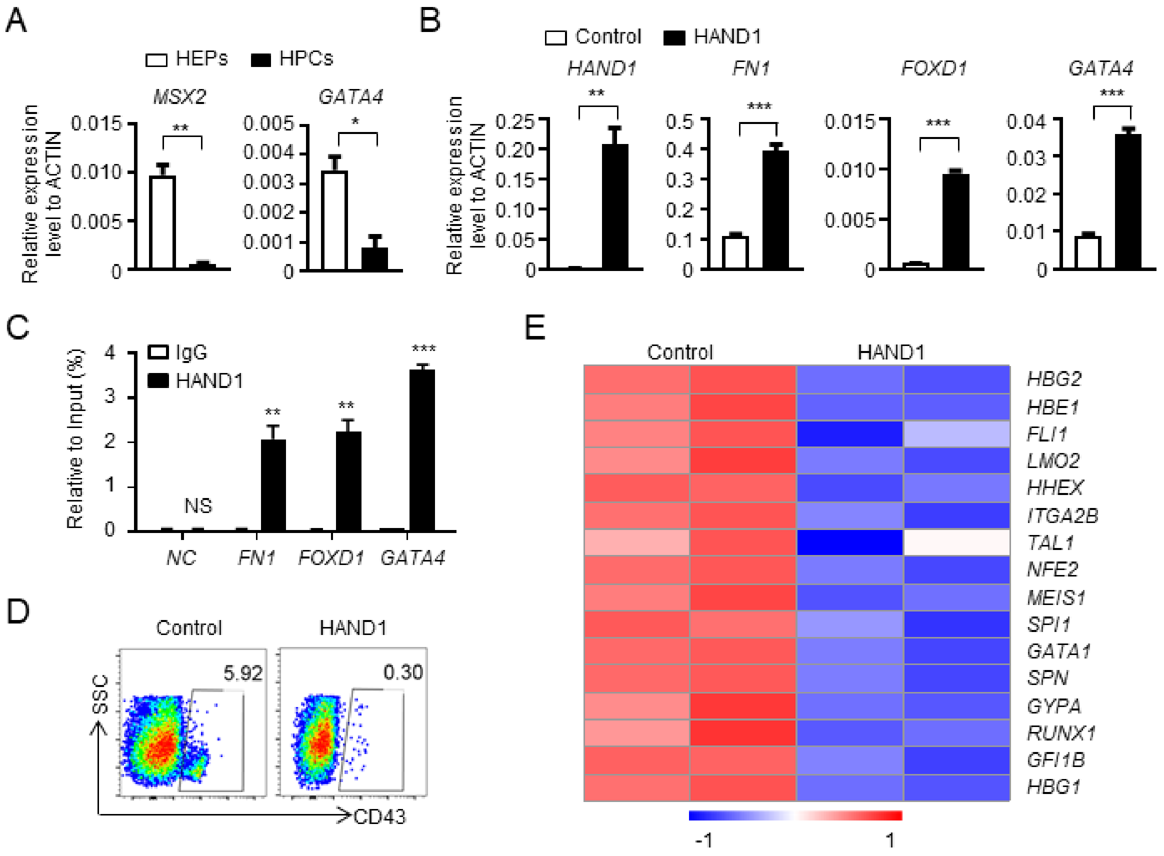
A
HEPs HPCs
MSX2
GATA4
Relative expression level to ACTIN
B
Control HAND1
HAND1
FN1
FOXD1
GATA4
C
IgG
HAND1
Relative to Input (%)
NS
NC
FN1
FOXD1
GATA4
D
Control
HAND1
5.92
0.30
SSC
CD43
E
Control
HAND1
HBG2
HBE1
FLI1
LMO2
HHEX
ITGA2B
TAL1
NFE2
MEIS1
SPI1
GATA1
SPN
GYPA
RUNX1
GFI1B
HBG1
-1
1

Fig. S7

Figure. S7 HAND1 Mediates the Suppressive Function of TGF-β Signaling in EHT. Related to Figure 7

(A) The relative mRNA expression of *MSX2* and *GATA4* between HEPs and HPCs during hematopoietic differentiation of hESCs. (B) The relative mRNA expression of *HAND1* and representative mesenchymal genes with or without HAND1 overexpression during the EHT process. (C) ChIP-qPCR analysis of the occupancy of HAND1 at the promoter region of *FN1*, *FOXD1* and *GATA4*. (D) Flow cytometry analysis of the percentage of $CD43^+$ HPCs with or without HAND1 overexpression. (E) The heatmap of hematopoiesis-associated genes with or without HAND1 overexpression. NS, not significant, *P<0.05, **P<0.01 and ***P<0.001.

Supplemental Experimental Section

Flow Cytometry

Accutase was used to dissociate differentiated cells into single cells. Then single cells were washed with PBS and centrifuged. After centrifugation, cells were resuspended with 100 ul 0.2% BSA to incubate with corresponding antibodies and isotype at 4℃ for 25-30 min. Prior to flow cytometry analysis, cells were washed with PBS and resuspended with 300 ul PBS containing DAPI (1 ug). Canto II was used to perform flow cytometry analysis, and Flowjo V10 was used for analyzing results. Detailed information on antibodies is listed in Supplementary Table S2.

Cell Sorting and MACS

For cell sorting, cells were dissociated using Accutase into single cells and washed with PBS to remove Accutase. Cells were then resuspended into 500 ul sterile 0.2% BSA supplemented with antibodies for 30 min at 4℃. After washes with PBS, cells were sorted using ARIA (BD, USA). Detailed information on antibodies is listed in Supplementary Table S2. For MACS, every $1x10^8$ cells were resuspended in 300 ul sorting buffer supplemented with 100 ul blocking buffer and 100 ul microbeads. After incubating at 4℃ for 30 min, cells were washed with PBS to remove redundant microbeads. Then cells were loaded into the column which was placed in the magnetic field. After the passage of unlabeled cells through the column, the column was placed on a collection tube to collect the labeled cells.

Immunofluorescence

Cells were washed with PBS for 3 times to remove redundant medium and fixed with 4% PFA at room temperature for 20 min. After fixation, cells were washed with PBS 3 times and permeabilized with 0.2% Triton X-100 at room temperature for 20 min. Next, cells were washed using PBS for 3 times and blocked by 0.5% BSA for 1 hour. Finally, cells were incubated with antibodies at 4℃ overnight. After being stained with DAPI, images were collected by the use of Leica SP2 microscope. Detailed information on antibodies is listed in Supplementary Table S3.

Generation of Knockout hESC Lines Using CRISPR/Cas9

The small-guide RNAs targeting SNAI1 and HAND1 gene were designed utilizing the CRISPR Design Tool (http://tools.genome-engineering.org). The corresponding oligonucleotides were cloned into the CRISPR-Cas9-Lenti-V2 vector. To establish gene knockout hESC lines, lentivirus carrying sgRNA was infected into H1 cell line. After 2 weeks of selection with puromycin (1 μg/ml), cells were dissociated using Accutase into single cells, and then single-cell clones were selected and expanded for gene sequencing to identify gene knockout hESC lines. Detailed information of sgRNAs and genotyping primers are listed in Supplementary Table S4.

Establishing SNAI1 or HAND1-overexpressing hESC Lines

The coding region of SNAI1 or HAND1 carrying the FLAG tag was synthesized by Youbio Company (http://www.youbio.cn/). The nucleotide fragments were cloned into SpeI and BmtI double-digested pBASE vector for further experiments. To establish SNAI1 or HAND1 overexpressing hESC lines, lentivirus carrying the constructed vector was infected into the

H1 cell line. Then cells were subsequently selected with puromycin (1 μg/ml) and determined with flow cytometry and real-time PCR.

qRT-PCR

qRT-PCR was performed as previously described.[1] The detailed primer sequences are shown in Supplemental Table S5.

Western blotting

Western blotting was performed as previously described.[1] The detailed information of antibodies used is shown in Supplemental Table S3.

ChIP-qPCR

The cells derived from SNAI1 or HAND1-Overexpressing H1 hESCs were collected for ChIP-qPCR assay. ChIP assay was carried out using the Magna ChIP^{TM} A/G kit (Millipore) following the manufacturer's suggestions. The detailed information of antibodies used is shown in Supplemental Table S3. And the primers used in ChIP-qPCR were listed in Supplementary Table S6.

Supplementary Table S1.

Signaling Pathway Small Molecules Used in This Study.

Reagent	Cat. NO.	Finally Concentration	Source
IWP2	S7085	3uM	SELLECK
XAV939	S1180	5uM	SELLECK
CHIR99021	S1263	3uM	SELLECK
BIO	S7198	2uM	SELLECK
SB431542	S1067	20uM	SELLECK
Repsox	S7223	1uM	SELLECK
Rimonabant	S3021	2.5uM	SELLECK
AM251	S2819	1uM	SELLECK

Supplementary Table S2.

Flow Cytometry Antibodies Used in This Study.

Antigen	Fluorochrome conjugated	Source	Cat#
APLNR	APC	R&D systems	FAB856A
CD31	PE	BD bioscience	555446
CD31	APC	eBioscience	17-0319-42
CD31	FITC	BD bioscience	555445
CD34	APC	BD bioscience	555824
CD34	PE	BD bioscience	555822
CD43	PE	BD bioscience	560199
CD43	APC	BD bioscience	560198
CD43	PerCP-eFluor 710	eBioscience	46-0438-42
CD45	PE	BD bioscience	560975

Supplementary Table S3.

The Sources and Dilutions of the Antibodies Used in This Study.

(I: Immunofluorescence; W: Western Blotting; C: ChIP)

Antibody	Source	Cat#	Dilution
OCT3/4	Santa Cruz	SC-9081	1:500(I)
SOX2	Millipore	AB5603	1:200(I)
NANOG	Cell Signaling	#3580	1:200(I)
CD43	Santa Cruz	sc-51727	1:200(I)
DAPI	Solarbio	C0060	1:1000(I)
p-SMAD2/3	CST	8828S	1:500(W)
β-Catenin	CST	9562S	1:1000(W)
Alpha-tubulin	Abcam	Ab11304	1:2000(W)
Histone H3	CST	2650S	1:1000(W)
Flag	Sigma	F1804	2μg (C)

Supplementary Table S4.

The Primer Sequences Used for sgRNA Synthesis and the Amplification of Target Sequence Fragments (TSF)

Primer	Sequence 5’-3’
SNAI1 E1g2 F	GTTAGGCTTCCGATTGGGGT
SNAI1 E1g2 R	ACCCCAATCGGAAGCCTAAC
SNAI2 E1g1 F	GCGGTAGTCCACACAGTGAT
SNAI2 E1g1 R	ATCACTGTGTGGACTACCGC
ZEB2 E1g1 F	GTTTGCGCCTCTTGCACCGG
ZEBI2 E1g1 R	CCGGTGCAAGAGGCGCAAAC
SNAI1 TSF F	GAGTACTTAAGGGAGTTGGCGG
SNAI1 TSF R	GTATTGAGAATCGGCCCCAC
SNAI2 TSF F	GTAACTCCGTAGGTGCCAG
SNAI2 TSF R	GCTACTTCCTGACTACTC
ZEB2 TSF F	CTCGCGGGTGCAACACACC
ZEBI2 TSF R	GGGTGCCTGACGCTCAC

Supplementary Table S5.

qRT-PCR (quantitative real time polymerase chain reaction) Primers Used in This Study.

Primer	Sequence 5'-3'
hACTIN-F	CTCTTCCAGCCTTCCTTCCT
hACTIN-R	AGCACTGTGTGTTGGCGTACAG
hBMP2-F	CGTGACCAGACTTTTGGACAC
hBMP2-R	GGCATGATTAGTGGAGTTCAG
hCDH1-F	TGCCCAGAAAATGAAAAAGG
hCDH1-R	GTGTATGTGGCAATGCGTTC
hEPCAM-F	GCGTTCGGGCTTCTGCTTGC
hEPCAM-R	CCGCTCTCATCGCAGTCAGGA
hFN1-F	TGACCCCTACACAGTTTCCCA
hFN1-R	TGATTCAGACATTCGTTCCCAC
hFOXD1-F	ATGACCCTGAGCACTGAGAT
hFOXD1-R	CCTCTTCCTCGTCTTCTT

hGATA1-F	GACAGGACAGGCCACTACCT
hGATA1-R	CTGCCCGTTTACTGACAATC
hGATA4-F	CGACACCCCAATCTCGATATG
hGATA4-R	GTTGCACAGATAGTGACCCGT
hHAND1-F	CACCAAGCTCTCCAAGATCA
hHAND1-R	GCGCCCTTTAATCCTCTTCT
hMSX2-F	TTACCACATCCCAGCTCCTC
hMSX2-R	CCTGGGTCTCTGTGAGGTTC
hNANOG-F	TTTGTGGGCCTGAAGAAAACT
hNANOG-R	AGGGCTGTCCTGAATAAGCAG
hSNAI1-F	AATCGGAAGCCTAACTACAGCG
hSNAI1-R	GTCCCAGATGAGCATTGGCA
hSNAI2-F	CGAACTGGACACACATACAGTG
hSNAI2-R	CTGAGGATCTCTGGTTGTGGT
hNRP1-F	ACGATAAATGTGGCGATACT
hNRP1-R	CCAACAGCCTTGAATGCACT

hOCLN-F	ATTGCCATCTTTGCCTGTGTGG
hOCLN-R	GCCATAGCCATAGCCACTTCCG
hPOU5F1-F	CTTGAATCCCGAATGGAAAGGG
hPOU5F1-R	GTGTATATCCCAGGGTGATCCTC
hSOX2-F	GCCGAGTGGAAACTTTTGTCG
hSOX2-R	GGCAGCGTGTACTTATCCTTCT
hSOX7-F1	CCTCTCCTTCTTGTGCCTTG
hSOX7-R1	GGGAGGAAAGCTGGTGTG
hMESP1-F	AGCCCAAGTGACAAGGGACAACT
hMESP1-R	AAGGAACCACTTCGAAGGTGCTGA
hTBX20-F	CAAGCCCCAACTCTCCTCTC
hTBX20-R	CTCCACCAAACTCCCCATGA
hZEB2-F	GCCGAGTGGAAACTTTTGTCG
hZEB2-R	GGCAGCGTGTACTTATCCTTCT
hAXIN2-F	CTGGTGCAAAGACATAGCCA
hAXIN2-R	AGTGTGAGGTCCACGGAAAC

hLEFTY-F	CAGCTGGAGCTGCACACCCT
hLEFTY-R	TCCTGGTACCCTCGAACA

Supplementary Table S6.

ChIP-qPCR (chromatin immunoprecipitation-quantitative polymerase chain reaction) Primers Used in This Study.

Primer	Sequence 5'-3'
SNAI1 NC-F	GCTTCTCAGTACGGACTCGC
SNAI1 NC-R	GCCTGGGCTCTTGCCTTCA
SNAI1 SNAI2-F	CTCTGACAAGTCTTGACAT
SNAI1 SNAI2-R	GAATAAGGAAAGAACAAAT
SNAI1 ZEB2-F	TCCAGTCCAGAAATTCATC
SNAI1 ZEB2-R	TTTAGGTACCAGAGCCAGA
SNAI1 NRP1-F	GGAGAAGACAGGCTTGACCCG
SNAI1 NRP1-R	CTGGGAGCAGTGACGTGCG
SNAI1 TBX20-F	CCACTCAATACAAAGAAATG
SNAI1 TBX20-R	TTTGGTTATTAAAGTCCCC
SNAI1 BMP2-F	GTAGTCAGGCTTCCTCCAA
SNAI1 BMP2-R	GGATTAGCAACCCACAGAG

HAND1 NC-F	TACTACCGCTGTCAATCAC
HAND1 NC-R	ATAGCCTTTATGGGTCACT
HAND1 FN1-F	CGGAACTCCCGGTACTTAG
HAND1 FN1-R	CTCCCTGTTCGGACTTCTT
HAND1 FOXD1-F	AGAATGGGTTAAAGGGTCAA
HAND1 FOXD1-R	CCTCCAGATCGGGCGAGCG
HAND1 GATA4-F	AGTTCTGTTCCCACCATAT
HAND1 GATA4-R	AGTGAGATCCCATCTCAAA

Supplemental References

[1] H. Wang, C. Liu, X. Liu, M. Wang, D. Wu, J. Gao, P. Su, T. Nakahata, W. Zhou, Y. Xu, L. Shi, F. Ma, J. Zhou, *Stem Cell Reports*, **2018**, *10,* 447-460.
